# Lifestyle, Quality of Life, and Health

**DOI:** 10.1100/tsw.2003.60

**Published:** 2003-08-22

**Authors:** Soren Ventegodt, Joav Merrick

**Affiliations:** The Quality of Life Research Center, Copenhagen K, Denmark

**Keywords:** quality of life, QOL, lifestyle, smoking, alcohol, diet, exercise, health, prevention, public health, Denmark

## Abstract

This study was performed in order to investigate the connection between lifestyle, quality of life, and health status by way of a questionnaire-based survey. The questionnaire was mailed in February 1993 to 2,460 persons aged between 18 and 88, randomly selected from the CPR (Danish Central Register), and 7,222 persons from the Copenhagen Perinatal Birth Cohort 1959–61. A total of 1,501 persons between the ages of 18 and 88 years and 4,626 persons between the ages of 31 and 33 years returned the questionnaire (response rates 61.0% and 64.1%, respectively). Variables investigated in this study were: alcohol consumption, tobacco consumption, type of diet, amount of exercise taken, quality of life, self-assessed health status, and number of medical complaints from which respondent suffered. The results showed that health has a stronger correlation to quality of life (r = 0.5, *p* < 0.0001) than it has to lifestyle (r = 0.2, *p* < 0.0001). It is concluded that preventable diseases could be more effectively handled through a concentrated effort to improve quality of life rather than through an approach that focuses solely on the factors that are traditionally seen to reflect an unhealthy lifestyle.

### INTRODUCTION

There is a general consensus that many of the diseases that plague the Western world (which are not the result of external factors such as starvation, micro-organisms, infection, or genetic defects) are lifestyle related and, as such, preventable through lifestyle changes. Thus, increasing time and effort is being spent on developing public health strategies to promote “healthy” lifestyles. However, it is not a simple task to identify and dispel the negative and unhealthy parts of our modern lifestyle even with numerous behavioural factors that can be readily highlighted harmful, like the use of alcohol, use of tobacco, the lack of regular exercise and a high fat, low fibre diet.

However, there is more to Western culture and lifestyle than these factors, and if we focus only on them we risk overlooking others. We refer to other large parts of our life, for instance, the way we think about and perceive life (our life attitudes, our perception of reality, and our quality of life) and the degree of happiness we experience through the different dimensions of our existence. These factors or dimensions can now, to some degree, be isolated and examined[1,2]. The medical sociologist Aaron Antonovsky (1923–1994) from Ben Gurion University in Beer-Sheva, who developed the salutogenic model of health and illness[3,4], discussed the dimension, “sense of coherence,” that is closely related to the dimension of “life meaning” as perhaps the deepest and most important dimension of quality of life[1,2,3,4]. Typically, the clinician or researcher, when attempting to reveal a connection between health and a certain factor, sides with only one of the possible dimensions stated above. A simple, one-dimensional hypothesis is then postulated, for instance, that cholesterol is harmful to circulation. Cholesterol levels are then measured, and manipulated, and ensuing changes to circulatory function monitored. The subsequent result may show a significant though small connection which supports the initial hypothesis, which in turn becomes the basis for implementing preventive measures, such as a change of diet. The multifactorial dimension is therefore often overlooked.

The purpose of this presentation was to use the results of a cross-sectional survey examining close to 10,000 Danes to show and compare the connections between lifestyle and health, and the various dimensions of quality of life and health.

### METHODS

#### Population 1 (RH)

The Copenhagen Perinatal Birth Cohort 1959–61[5,6,7,8] is a prospective longitudinal perinatal study that included all deliveries (over 20 weeks gestation, birthweight over 250 g) that took place at the University Hospital (Rigshospitalet) in Copenhagen, Denmark during the period of September 21, 1959 to December 21, 1961. The cohort consisted of 9,006 pregnant women, who delivered 8,820 live born infants and 362 stillborn or late abortions over 250 g. 170 women gave birth to twins, three to triplets, and the remainder were single births. The pregnant women were contacted and examined before delivery as early in pregnancy as possible. In order to evaluate and code the social, medical, and obstetrical information uniformly, all examinations were done by the same physician[5]. He also examined all women after delivery and coded information made by the women herself during pregnancy and also the information describing the events in the delivery room[7].

The infants were examined by one of three pediatricians on the first and fifth days. Physical and neurological examinations were conducted. Upon discharge the mothers received a questionnaire related to child development during the first year, which they brought to the hospital at the age of 1 year for a physical reexamination (a total of 8,425 children survived 28 days) [6,7,8]. Follow-up physical examinations took place at 3 and 6 years with developmental recording done by the mothers between examinations. At a later stage all school health records were retrieved and coded by one physician[9]. More than 1,000 factors relating to pregnancy, birth, and child development were collected on each child resulting in numerous scientific publications over the last 40 years.

In 1993 a new follow-up study was performed and 7,222 of the surviving children were identified (now aged between 31 and 33 years) and contacted with a nonanonymous questionnaire[1]. A written reminder to nonresponders was sent a month later resulting in 4,626 usable responses (f = 2,489, m = 2,131) corresponding to a response rate of 64.1%. The response rate for each individual question was typically a little lower. These 4,626 persons constitute population 1.

### Population 2(CPR)

Using the Danish National Register (CPR-register) 2,460 Danes between the ages of 18 and 88 years were chosen randomly and contacted with the same questionnaire[1]. Of the 2,460 contacted we received 1,501 responses, corresponding to a response rate of 61.0%.

### The Questionnaire on Quality of Life

The questionnaire used in the study, “Questionnaire for the Self Evaluation of Quality of Life” (SEQOL), contained 317 questions with 205 in an easy-to-answer multiple-choice format. The questionnaire was divided into the following sections: social information, lifestyle, illness, sexuality, self-perception, life perception, and eight series of questions measuring the quality of life[13].

The development of the questionnaire and progression of the study was guided by the following methodological demands for quantitative questionnaire-based quality of life research[13]:

1. A clear definition of the quality of life.
2. A philosophy of life based on the definition of point 1.
3. A theory that has this philosophy as its framework by
    a) deducing questions that are unambiguous, mutually exclusive, and together are fully exhaustive, and by
    b) establishing the relative weighting of each question.
4. A number of response options that might be given a quantitative interpretation on a fraction scale.
5. Technical checks in terms of reproducibility, sensitivity, well-scaledness, etc.
6. The survey must be meaningful to both researchers, respondents, and those who use the results.
7. An appreciation of the aesthetic dimension.

The present study followed these requirements[13] with the theoretical basis for quality of life measurement based upon the integrative quality of life theory[13]. It organises eight individual theories of quality of life into a spectrum ranging from subjective (self-evaluated) to objective (externally evaluated) quality of life and spanning a core of theories that consider quality of life as deriving from human nature or human existence itself (existential theories). These eight theories or dimensions of life quality were operationalized into eight rating scales, then grouped into three dimensions:

#### I. Subjective Dimensions

1. Immediate, self-experienced well-being
2. Satisfaction with life
3. Happiness

**II. Existential Dimensions**

4. Needs fulfillment
5. Subjective experience of objective temporal domains (family, work, leisure)
6. Subjective experience of objective spatial domains (satisfaction with social relationships)
7. Expression of life's potentials

**III. Objective Dimension**

8. Objective factors (income, employment, education, etc.)

Eighty-five of the questions in the questionnaire were used to measure quality of life along these eight dimensions. A Likert-scale with five response options was symmetrically arranged around a neutral midpoint. As an example, well-being was measured by the question, "How are you feeling now?", and the response options given were "very good," "good," "neither good nor poor," "poor," and "very poor." The central and precisely worded mid-point (neither good nor poor), the response options symmetrically aligned up and down the scale (good, poor) and the use of the same amplifier (very) all combine to suggest that the five points on the scale may be considered equidistant.

If an underlying scale was selected ranging from 0 to 100%, from the worst imaginable to the best imaginable quality of life, the five response options may be reasonably positioned at 10, 30, 50, 70, and 90%. In other words, if a respondent checks "good," his or her well-being is measured as 70%. In this manner, an approximated ratio scale was obtained, so that means could be computed and compared.

A weighted mean for the eight quality of life dimensions was computed by subjective and existential measures, respectively. The resulting overall measure was global (covers all aspects of life, not merely health-related aspects) and generic (not disease-related or intended for a specific category of patients).

Significance levels for the relationships between each variable and the measured quality of life were computed for the continuous variable using classical correlation and a modified regression[13], while in the case of the discrete variables, every group was tested individually against the rest of the sample HO: $\mu i = \mu non\text{-}i$ (that is, the null hypothesis that the mean quality of life for a particular group [for example, smokers]) was significantly different from the mean quality of life for the rest of the population [for example, those that do not smoke]).

The questionnaire has been validated[1,2,10,11], and the measurement instruments (the rating scales) proved to be valid and sensitive to the same degree as commonly recognised international instruments. A 1-month and a 3-month test-retest for reproducibility showed correlation coefficients for the eight instruments ranging from 0.6 to 0.9. A qualitative assessment of the validity of the questionnaire was performed, in which 80% of the respondents indicated that the questionnaire items expressed all dimensions relating to their quality of life, 17% were in doubt, and 3% felt they did not express all dimensions, which was found to be acceptable.

### The Questionnaire on Health in SEQOL

The following questions on health were used in the questionnaire:

**1. How do you rate your own physical health now?**

| | physical | mental |
|---|---|---|
| very good | 1 | 1 |
| good | 2 | 2 |
| neither good nor poor | 3 | 3 |
| poor | 4 | 4 |
| very poor | 5 | 5 |

**2. Do you have any of the following health problems now? (Please circle a number in each row)**

| | No | Yes, somewhat | Yes, a lot |
|---|---|---|---|
| pain/discomfort in shoulder or neck? | 1 | 2 | 3 |
| pain/discomfort in back or buttocks? | 1 | 2 | 3 |
| pain/discomfort in arms, hands, legs, knees, hips, or joints? | 1 | 2 | 3 |
| headache? | 1 | 2 | 3 |
| a rapid heart beat? | 1 | 2 | 3 |
| nervousness, restlessness, or anxiety? | 1 | 2 | 3 |
| stress? | 1 | 2 | 3 |
| difficulty sleeping or insomnia? | 1 | 2 | 3 |
| melancholy, depression, or unhappiness? | 1 | 2 | 3 |
| tiredness? | 1 | 2 | 3 |
| stomach pain or stomachache? | 1 | 2 | 3 |
| indigestion, diarrhea, or constipation? | 1 | 2 | 3 |
| eczema, rash, or itching? | 1 | 2 | 3 |
| cold, head cold, or cough? | 1 | 2 | 3 |
| difficulty in breathing or breathlessness? | 1 | 2 | 3 |
| discomfort in the sexual organs (f)? | 1 | 2 | 3 |

### The Questionnaire on Lifestyle in SEQOL

The following questions were used in the questionnaire to assess lifestyle:

1. **Your eating habits? (Please circle the lines that relate most closely to you)**

1 I eat what I like
2 I think I have a healthy diet
3 I think I have an unhealthy diet
4 I have a low calorie diet because of my weight
5 I eat ready-prepared dishes/fast food at least once a week
6 I eat vegetarian food
7 Information campaigns influence my eating habits

2. **How many hours of exhausting exercise do you get a week? (Possibly during work)**

1 0 hours
2 about 30 minutes
3 about 1 hour
4 about 2 hours
5 about 4 hours or more

**3. How much alcohol did you drink in total last week (i.e., Monday, Tuesday, Wednesday, and Thursday)?**

Ordinary beer (number of pints): ____
Strong beer (number of pints) : ____
Red or white wine (number of glasses): ____
Dessert wine (number of glasses): ____
Spirits (number of drinks): ____

4. **How much alcohol did you drink in total last weekend (i.e., Friday, Saturday, and Sunday <u>inclusive</u>)?**

Ordinary beer (number of pints): ____
Strong beer (number of pints) : ____
Red or white wine (number of glasses): ____
Dessert wine (number of glasses): ____
Spirits (number of drinks): ____

5. **Was the past week typical of your normal level of alcohol consumption?**

1 yes
2 no, more than typical
3 no, less than typical

6. **Do you smoke?**

1 Yes, daily
2 Yes, now and then
3 No, I stopped less than a year ago
4 No, I stopped more than a year ago
5 No, I have never smoked

7. ***If yes*, how much do you smoke on average?**

Number of cigarettes daily ____
Number of cheroots daily ____
Number of cigars daily ____
Amount of smoking tobacco (grams) ____

## RESULTS

### Health against Lifestyle

For both populations 1 and 2 we found only a very modest connection between health — in all dimensions — and lifestyle. We conducted two analyses: a classical correlation analysis (Table 1) and a regression analysis (Table 2) termed "weighted linear regression", which compensated for the number of respondents in the different groups[1].

From the tables we see that where we have significant results only a very small connection between lifestyle and health existed.

#### TABLE 1
### Pearson Correlation (r, p) between Lifestyle Factors and Health

| | Self-Evaluated Physical Health | | | | Self-Evaluated Mental Health | | | | Satisfaction with Health | | | |
|---|---|---|---|---|---|---|---|---|---|---|---|---|
| | CPR | | RH | | CPR | | RH | | CPR | | RH | |
| | (r) | (p) | (r) | (p) | (r) | (p) | (r) | (p) | (r) | (p) | (r) | (p) |
| Alcohol consumption | 0.0496 | 0.0884 | 0.0124 | 0.4537 | -0.0172 | 0.5671 | -0.3730 | 0.0261 | 0.0389 | 0.1825 | 0.0158 | 0.3370 |
| Smoking | -0.0604 | 0.0205 | 0.0799 | 0.0001 | -0.0311 | 0.2461 | 0.0746 | 0.0001 | -0.0524 | 0.0446 | -0.0970 | 0.0001 |
| Exercise | 0.1209 | 0.0001 | 0.1495 | 0.0001 | 0.0589 | 0.0284 | 0.0926 | 0.0001 | 0.0898 | 0.0014 | 0.1225 | 0.0001 |
| Diet* (Healthy/unhealthy) | -0.0046 | 0.8632 | 0.0459 | 0.0020 | 0.0153 | 0.0586 | 0.0343 | 0.0226 | 0.0108 | 0.6835 | 0.0279 | 0.0596 |
| Health/unhealthy lifestyle | 0.0886 | 0.0006 | 0.1433 | 0.0001 | 0.0641 | 0.0163 | 0.1241 | 0.0001 | 0.0778 | 0.0028 | 0.1309 | 0.0001 |

| | Number of Complaints | | | | Overall Health Status | | | |
|---|---|---|---|---|---|---|---|---|
| | CPR | | RH | | CPR | | RH | |
| | (r) | (p) | (r) | (p) | (r) | (p) | (r) | (p) |
| Alcohol consumption | 0.0658 | 0.0230 | 0.0513 | 0.0019 | 0.0315 | 0.2981 | 0.0068 | 0.6934 |
| Smoking | -0.1177 | 0.0001 | -0.1815 | 0.0001 | -0.0740 | 0.0061 | -0.1189 | 0.0001 |
| Exercise | 0.0751 | 0.0040 | 0.1054 | 0.0001 | 0.1124 | 0.0001 | 0.1489 | 0.0001 |
| Diet* (Healthy/unhealthy) | 0.0315 | 0.2310 | 0.0405 | 0.0062 | 0.0290 | 0.2899 | 0.0557 | 0.0003 |
| Health/unhealthy lifestyle | 0.0823 | 0.0021 | 0.1503 | 0.0001 | 0.1073 | 0.0001 | 0.1684 | 0.0001 |

* Healthy diet was determined by the following factors: fat/low fat, fast-food/not fast-food, vegetarian/not vegetarian, and self evaluated as healthy / unhealthy by the respondent.
CPR = Sample randomly drawn from the Danish Central Persons Register.
RH= Sample taken from Rigshospitalet (The Copenhagen Perinatal Birth Cohort 1959-61 at University Hospital)

**TABLE 2**
**Connection between Lifestyle and Health Expressed through Weighted Linear Regression (a, p)**

| | Self-Evaluated Physical Health | | | | Self-Evaluated Mental Health | | | | Satisfaction with Health | | | |
|---|---|---|---|---|---|---|---|---|---|---|---|---|
| | CPR | | RH | | CPR | | RH | | CPR | | RH | |
| | (a) | (p) | (a) | (p) | (a) | (p) | (a) | (p) | (a) | (p) | (a) | (p) |
| Alcohol consumption | -2.7 | ±9.7 | -11.1 | ±5.0 | -2.9 | ±9.3 | -12.1 | ±5.8 | -8.7 | ±9.1 | -7.4 | ±5.1 |
| Smoking | -13.9 | ±10.3 | -20.1 | ±10.5 | -4.0 | ±10.6 | -10.7 | ±11.5 | -6.1 | ±9.7 | -11.4 | ±10.5 |
| Exercise | 7.5 | ±3.5 | 10.0 | ±1.9 | 3.7 | ±3.6 | 6.8 | ±2.2 | 5.0 | ±3.4 | 8.4 | ±2.0 |
| Diet** (Healthy/unhealthy) | 6.1 | ±7.6 | 5.1 | ±5.1 | 7.4 | ±8.2 | 3.9 | ±5.7 | 4.0 | ±7.7 | 7.5 | ±5.1 |
| Health/unhealthy lifestyle | 13.8 | ±8.6 | 18.8 | 4.9 | 12.5 | ±8.8 | 18.0 | ±5.4 | 12.7 | ±8.1 | 15.7 | ±4.9 |

| | Number of Complaints | | | | Overall Health Status | | | |
|---|---|---|---|---|---|---|---|---|
| | CPR | | RH | | CPR | | RH | |
| | (a) | (p) | (a) | (p) | (a) | (p) | (a) | (p) |
| Alcohol Consumption | 1.2 | ±5.6 | 6.2 | ±3.5 | -5.4 | ±6.6 | -8.9 | ±3.8 |
| Smoking | -5.2 | ±6.3 | -16.3 | ±6.9 | -9.6 | ±7.3 | -14.0 | ±7.5 |
| Exercise | 2.8 | ±2.1 | 5.0 | ±1.3 | 4.8 | ±2.4 | 7.2 | ±1.4 |
| Diet** (Healthy/unhealthy) | 1.6 | ±4.9 | 2.4 | ±3.4 | 4.0 | ±5.7 | 5.2 | ±3.7 |
| Health/unhealthy Lifestyle | 7.7 | ±5.0 | 11.9 | ±5.8 | 11.9 | ±5.8 | 15.3 | ±3.6 |

**a is the difference in health between the highest and lowest quality of life group as a percentage of the average health for all groups. $p = \pm 1.96 \times SE\{a\}$.
** Healthy diet was determined by the following factors: fat/low fat, fast-food/not fast-food, vegetarian/not vegetarian, and self evaluated as healthy / unhealthy by the respondent.
CPR = Sample randomly drawn from the Danish Central Persons Register.
RH= Sample taken from Rigshospitalet (The Copenhagen Perinatal Birth Cohort 1959-61 at University Hospital)

### Health against Quality of Life

Using the same regression and correlation tests as above, we found large and significant connections between the dimensions of health and quality of life, both in the CPR study and in the cohort (see Tables 3 and 4).

The analysis showed a large and clear connection between quality of life and health.

### TABLE 3
### Pearson Correlation (r,p) between Health and Quality of Life

| | Self-Evaluated Physical Health | | | | Self-Evaluated Mental Health | | | | Satisfaction with Health | | | |
|---|---|---|---|---|---|---|---|---|---|---|---|---|
| | CPR | | RH | | CPR | | RH | | CPR | | RH | |
| | (r) | (p) | (r) | (p) | (r) | (p) | (r) | (p) | (r) | (p) | (r) | (p) |
| Well-being | 0.5020 | 0.0001 | 0.4687 | 0.0001 | 0.6595 | 0.0001 | 0.7052 | 0.0001 | 0.5230 | 0.0001 | 0.4699 | 0.0001 |
| Life satisfaction | 0.3812 | 0.0001 | 0.3667 | 0.0001 | 0.6217 | 0.0001 | 0.6531 | 0.0001 | 0.4369 | 0.0001 | 0.3971 | 0.0001 |
| Happiness | 0.2785 | 0.0001 | 0.3034 | 0.0001 | 0.5162 | 0.0001 | 0.5753 | 0.0001 | 0.3579 | 0.0001 | 0.3322 | 0.0001 |
| Fulfillment of needs (approx. Maslow) | 0.2457 | 0.0001 | 0.3185 | 0.0001 | 0.4888 | 0.0001 | 0.5155 | 0.0001 | 0.2838 | 0.0001 | 0.3117 | 0.0001 |
| Temporal domains* | 0.2886 | 0.0001 | 0.3454 | 0.0001 | 0.5263 | 0.0001 | 0.5451 | 0.0001 | 0.3206 | 0.0001 | 0.3647 | 0.0001 |
| Spatial domains** | 0.3023 | 0.0001 | 0.3530 | 0.0001 | 0.4560 | 0.0001 | 0.4643 | 0.0001 | 0.4048 | 0.0001 | 0.4378 | 0.0001 |
| Expression of life's potential*** | 0.2274 | 0.0001 | 0.2400 | 0.0001 | 0.3264 | 0.0001 | 0.4062 | 0.0001 | 0.2337 | 0.0001 | 0.2457 | 0.0001 |
| Objective factors | 0.1745 | 0.0001 | 0.1910 | 0.0001 | 0.2142 | 0.0001 | 0.2907 | 0.0001 | 0.1714 | 0.0001 | 0.1892 | 0.0001 |
| Overall QL | 0.3749 | 0.0001 | 0.3722 | 0.0001 | 0.5232 | 0.0001 | 0.5903 | 0.0001 | 0.4175 | 0.0001 | 0.3899 | 0.0001 |

| | Number of Complaints | | | | Overall Health Status | | | |
|---|---|---|---|---|---|---|---|---|
| | CPR | | RH | | CPR | | RH | |
| | (r) | (p) | (r) | (p) | (r) | (p) | (r) | (p) |
| Well-being | 0.4513 | 0.0001 | 0.4710 | 0.0001 | 0.6815 | 0.0001 | 0.6830 | 0.0001 |
| Life satisfaction | 0.3876 | 0.0001 | 0.4182 | 0.0001 | 0.5800 | 0.0001 | 0.5855 | 0.0001 |
| Happiness | 0.2775 | 0.0001 | 0.3469 | 0.0001 | 0.4634 | 0.0001 | 0.4991 | 0.0001 |
| Fulfillment of needs (approx. Maslow) | 0.2749 | 0.0001 | 0.3240 | 0.0001 | 0.4191 | 0.0001 | 0.4736 | 0.0001 |
| Temporal domains* | 0.0307 | 0.0001 | 0.3485 | 0.0001 | 0.4722 | 0.0001 | 0.5162 | 0.0001 |
| Spatial domains** | 0.2848 | 0.0001 | 0.3158 | 0.0001 | 0.4694 | 0.0001 | 0.5065 | 0.0001 |
| Expression of life's potential*** | 0.1927 | 0.0001 | 0.2106 | 0.0001 | 0.3170 | 0.0001 | 0.3479 | 0.0001 |
| Objective factors | 0.2197 | 0.0001 | 0.2480 | 0.0001 | 0.2275 | 0.0001 | 0.2843 | 0.0001 |
| Overall QL | 0.3621 | 0.0001 | 0.3988 | 0.0001 | 0.5235 | 0.0001 | 0.5588 | 0.0001 |

*Family, work, and leisure time.
**Self, others, world.
***Personal relations to all known persons, self, and surrounding world.
CPR = Sample randomly drawn from the Danish Central Persons Register.
RH= Sample taken from Rigshospitalet (The Copenhagen Perinatal Birth Cohort 1959-61 at University Hospital)

**TABLE 4**
**The Connection between Quality of Life and Health Expressed through Weighted Linear Regression (a, p)**

| | Self-Evaluated Physical Health | | | | Self-Evaluated Mental Health | | | | Satisfaction with Health | | | |
|---|---|---|---|---|---|---|---|---|---|---|---|---|
| | CPR | | RH | | CPR | | RH | | CPR | | RH | |
| | (a)* | (p) | (a) | (p) | (a) | (p) | (a) | (p) | (a) | (p) | (a) | (p) |
| Well-being | 60.8 | ±10.3 | 57.4 | ±6.1 | 0.6595 | ±10.5 | 94.7 | ±5.4 | 58.0 | ±9.9 | 59.4 | ±6.1 |
| Life satisfaction | 37.2 | ±10.5 | 48.1 | ±5.5 | 80.3 | ±9.4 | 83.7 | ±4.9 | 51.2 | ±10.4 | 49.2 | ±5.4 |
| Happiness | 39.1 | ±14.4 | 44.5 | ±6.9 | 87.4 | ±12.8 | 95.9 | ±6.5 | 49.8 | ±13.6 | 45.9 | ±6.8 |
| Fulfillment of needs (approx. Maslow) | 24.3 | ±8.7 | 50.4 | ±14.9 | 64.1 | ±8.1 | 68.3 | ±15.6 | 28.6 | ±8.4 | 39.4 | ±14.8 |
| Temporal domains | 27.9 | ±11.1 | 60.6 | ±14.9 | 66.7 | ±10.5 | 87.2 | ±14.3 | 43.5 | ±10.6 | 71.9 | ±14.7 |
| Spatial domains** | 25.2 | ±16.4 | 42.1 | ±5.6 | 73.5 | ±15.8 | 64.9 | ±5.7 | 58.5 | ±15.3 | 56.9 | ±5.4 |
| Expression of life's potential† | 25.4 | ±9.6 | 25.3 | ±7.2 | 32.6 | ±10.2 | 42.8 | ±8.0 | 28.6 | ±9.2 | 25.5 | ±7.3 |
| Objective factors | 22.7 | ±16.6 | 36.7 | ±11.9 | 17.1 | ±19.0 | 51.2 | ±12.6 | 18.3 | ±16.3 | 37.1 | ±12.0 |
| Overall QL | 50.7 | ±7.6 | 46.7 | ±4.8 | 71.7 | ±7.9 | 78.0 | ±4.5 | 55.9 | ±7.6 | 47.8 | ±4.8 |

| | Number of Complaints | | | | Overall Health Status | | | |
|---|---|---|---|---|---|---|---|---|
| | CPR | | RH | | CPR | | RH | |
| | (a) | (p) | (a) | (p) | (a) | (p) | (a) | (p) |
| Well-being | 39.9 | ±6.3 | 48.9 | ±4.0 | 0.7 | ±6.2 | 62.7 | ±3.6 |
| Life satisfaction | 31.0 | ±6.0 | 42.1 | ±3.6 | 48.0 | ±6.3 | 53.6 | ±3.5 |
| Happiness | 38.6 | ±8.0 | 44.5 | ±4.5 | 53.4 | ±8.9 | 56.1 | ±4.6 |
| Fulfillment of needs (approx. Maslow) | 24.1 | ±5.3 | 45.3 | ±9.9 | 34.2 | ±5.7 | 53.5 | ±10.6 |
| Temporal domains* | 30.6 | ±6.8 | 36.7 | ±9.9 | 41.3 | ±7.1 | 62.4 | ±9.6 |
| Spatial domains** | 27.3 | ±9.9 | 28.0 | ±3.9 | 44.5 | ±10.3 | 47.0 | ±3.9 |
| Expression of life's potential† | 12.9 | ±5.6 | 11.8 | ±5.1 | 22.0 | ±6.7 | 22.5 | ±5.4 |
| Objective factors | 10.9 | ±9.7 | 21.6 | ±7.6 | 15.9 | ±11.1 | 35.6 | ±8.2 |
| Overall QL | 27.7 | ±4.8 | 36.4 | ±3.2 | 47.2 | ±5.0 | 50.3 | ±3.2 |

a is the difference in health between the highest and lowest quality of life group as a percentage of the average health for all groups. $p = \pm 1.96 \times SE\{a\}$.
*Family, work, and leisure time.
**Self, others, world.
†Personal relations to all known persons, self, and surrounding world.
CPR = Sample randomly drawn from the Danish Central Persons Register.
RH= Sample taken from Rigshospitalet (The Copenhagen Perinatal Birth Cohort 1959-61 at University Hospital)

### Quality of Life against Lifestyle

To illustrate the above findings it may be worth having a look at the connections between health, quality of life and lifestyle (see Figure 1, Figure 2, and Figure 3). The important question is, What is the underlying factor responsible for determining the quality of life? Is it a simple and quite trivial connection to health status? Or, alternatively, is quality of life determined by numerous factors over which people have great influence. Is it reasonable to suggest that those with deep insight and knowledge about life can live their life in such a way that they thrive and maintain their health, while those who lack knowledge about life live in such a way that quality of life is not experienced and they become more susceptible to illness?

Of course this cannot be determined conclusively through a cross-sectional survey, and we continue the work prospectively. But the cross-sectional survey showed a strong connection between our quality of life and our personal relations — with partner, friends and work colleagues — and only a modest connection with objective factors such as annual income and level of education[12,13].

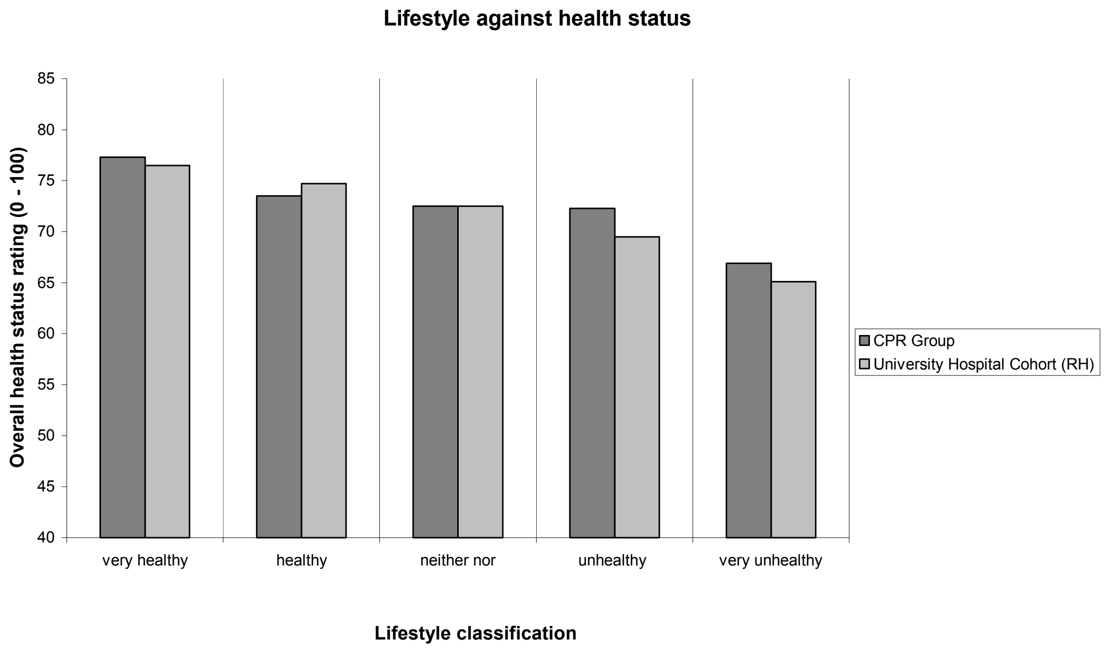

**Figure 1**. Lifestyle against health status. The statistical co-variation is about 10% in both studies. This is a far weaker co-variation than is seen between quality of life and health (figure 2), suggesting that global QOL is the most important factor of the two for preventing disease and improving public health.

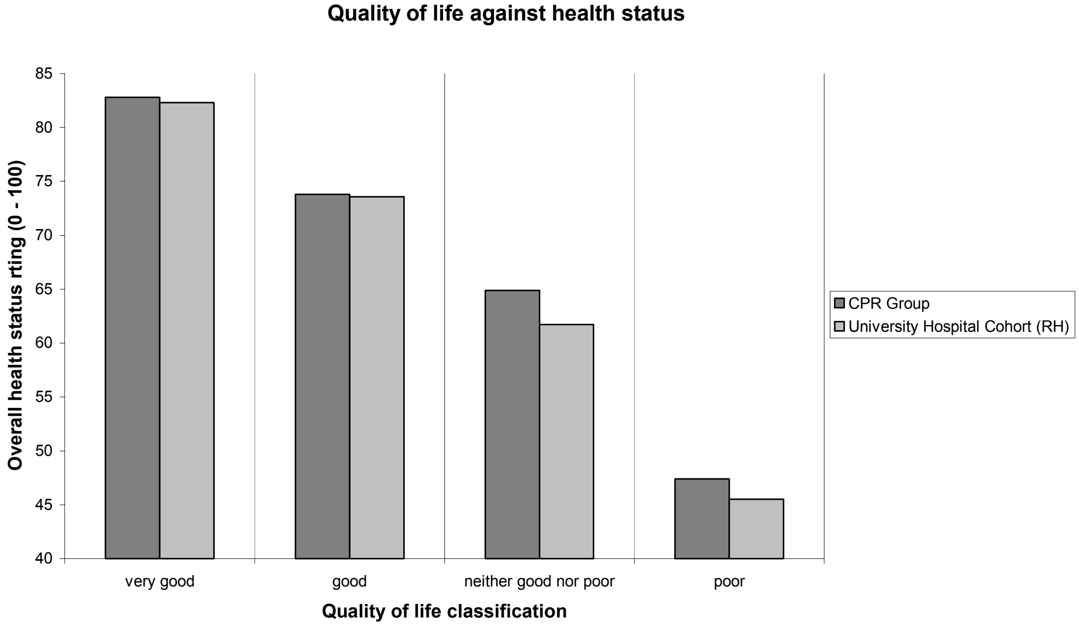

**Figure 2.** Global quality of life against health status. The statistical co-variation is about 40% in both studies. This is a far stronger co-variation than is seen between lifestyle and health (Figure 1), and between quality of life and lifestyle (Figure 3), suggesting that global QOL is the most important factor for preventing disease and improving health.

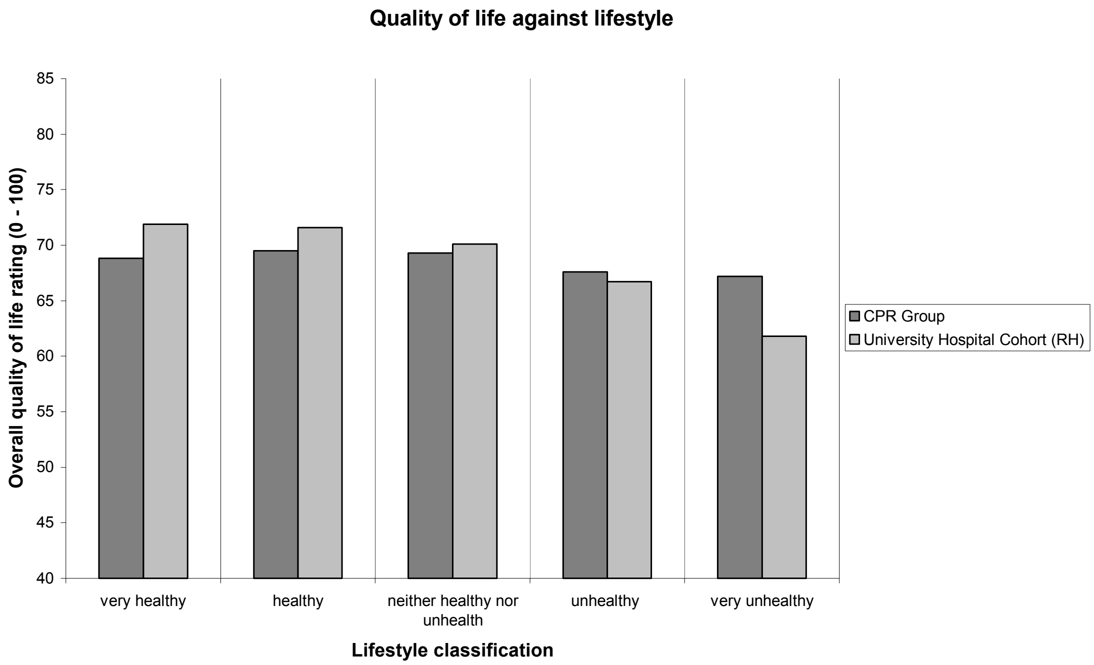

**Figure 3**: Global quality of life against lifestyle. The statistical co-variation is 5-10% in the two studies. This is a far weaker co-variation than is seen between quality of life and health (Figure 2). Taken together with the week connection between lifestyle and health status (Figure 1) the results suggests that lifestyle is only weakly connected to QOL and health. To improve QOL and health more than a change in lifestyle seems to be needed.

### DISCUSSION

The presented data seem to suggest that lifestyle factors have a much smaller connection with health than normally assumed in the medical world. On the other hand, quality of life, more specifically the self-evaluated experience of life, showed a very strong connection with self-evaluated health.

Numerous clinical trials have found links between lifestyle factors and health status, but perhaps the connection is an indirect one. Perhaps lifestyle is just an indicator of quality of life and it is quality of life that is the major determinant of health. When we controlled for quality of life we found, in general, no connection between a healthy/unhealthy lifestyle and self-evaluated health[13].

It has been demonstrated in the literature that psychotherapy treatments, in which the patient focuses on emotions and personal relationships, have positive effects on quality of life and survival[14,15,16], and that radical changes in life behaviours and attitudes can be beneficial to recovery[14]. There is also evidence to suggest that, in general populations, general well-being is positively related to life expectancy[17,18].

In view of these studies and our own results, it maybe reasonable to try and improve health by attempting to directly improve quality of life rather than just changing a particular aspect of someone's lifestyle, such as diet or exercise, as is often advised by physicians.

If a strong causal link between quality of life and health could be clearly demonstrated, it could perhaps be more effective for public health strategists to look at motivating populations to identify and improve the aspects of their lives with which they may be unhappy, such as their jobs or relationships.

By developing our understanding of the link between quality of life and health, greater steps may be made toward health maintenance and improvement. Seemingly, we lack a structured science that can integrate the concepts of quality of life, experience and consciousness into medical theory and clinical practice.

The lifestyle questionnaire is not validated. This is because of the objectivity of the data collected and the general agreement about the involved factors as the most important ones, and therefore we have not found validation necessary. A more severe problem with this study is that we measured health and QOL in very much the same way, while we measured lifestyle differently. This is a necessity, because the first two mentioned are subjective data, while the third consists of objective data. Health can be measured objectively in many dimensions such as lung capacity, physical strength, number of medical diagnosis or use of medication, but to measure global health subjective judgment is mandatory, either from a doctor or from the person himself. We believe that the data collected directly from the patient are the most valid. We have done our best to select reliable data and strongly believe that the presented correllations are not an artefact. Another critique could be raised against our scientific design, as we have chosen not to use the most advanced, but instead the simplest possible solutions all the way through the study in order not to get lost in the complex scenario; we use the motto, "the most simple questionnaire, the most simple strategies for statistical analysis." The statistical analysis could have been done much more elegantly using partial or even nonlinear regression, but we were convinced that the conclusion would have been very much the same, just wrought with all the uncertainty that inevitably comes from the use of advanced statistical procedures.

Even if more elaborated studies in the future show that we are wrong in our conclusion, it can never be harmful to encourage people to focus on improving their QOL and their human relations in general. We believe the good life can be lived when we feel good about ourselves and other people, and when we really enjoy the work we do.

### CONCLUSION AND QUESTIONS FOR FURTHER RESEARCH

The three most important things we learned from this study are:

- It seemed extremely important to be sharp about the concepts in use.
- We must discriminate between the concept of health itself — the state of body and mind including emotional and spiritual dimensions, the concept of lifestyle in medicine normally defined as health-related behaviour, and the concept of global QOL first the experience of life itself or, in a broader philosophical sense, the state of our existence as a whole.
- When we measure these three very different dimensions and look at weight of the statistical connections, it appears that the dimension of most importance for health is not lifestyle, but rather QOL. QOL and self-assessed physical and mental health are strongly correlated, while lifestyle is not strongly correlated to either QOL or health.

We therefore must encourage everybody involved with public health issues to be sharp and precise about their concepts, to include the concept of global QOL in the research, and to focus on improving QOL in the population, in order to improve the general public health.

Much more research is needed, and the most important question for further research seems to be how do we improve QOL in the aspects most relevant for health? We must also ask if we can actually use QOL as preventative or even curative medicine, in society as a whole, in the work place, and in the medical clinic [19].

### ACKNOWLEDGEMENTS

This paper reported results from the Danish Quality of Life Population Survey, the planning and execution of which would not have been possible without the aid of Dorte Loldrup Poulsen, Jørgen Hilden and Bengt Zachau-Christiansen. Funds were received from eleven Danish Foundations, including The 1991 Pharmacy, the Goodwill, the JL, E. Danielsens & Wife's, Emmerick Meyer's, the Frimodt-Heineken, the Family Hede Nielsen's, Petrus Andersen's, C.P. Frederiksen's and the Wedell-Wedellsborgs Foundations, and IMK Almene Fond. The research is approved by the Copenhagen Ethical Committee under numbers (KF) V 100.1762/90 and (KF) 01-502/93.

### BIOSKETCH

**Søren Ventegodt, MD,** is the director of the the Quality of Life Research Center in Copenhagen, Denmark. He is also responsible for a research clinic for holistic medicine in Copenhagen and used as a popular speaker throughout Scandinavia. He has published numerous scientific and popular articles and a number of books on holistic medicine, quality of life, and quality of working life. His most important scientific contributions are the comprehensive SEQOL questionnaire, the very short QoL5 questionnaire, the integrated QOL theory, the holistic process theory, the life mission theory, and the Danish Quality of Life Research Survey, 1991–94 in cooperation with the University Hospital of Copenhagen and the late pediatric professor Bengt Zachau-Christiansen. Website: www.livskvalitet.org/

**Joav Merrick, MD, DMSc,** is professor of child health and human development affiliated with the Zusman Child Development Center and Division of Community Health at the Ben Gurion University, Beer-Sheva, Israel and presently the medical director of the Division for Mental Retardation, Ministry of Social Affairs, Jerusalem and the director of the National Institute of Child Health and Human Development. He has authored and edited numerous publications in the fields of child and human development, rehabilitation, intellectual disability, disability, health, welfare, abuse, advocacy and prevention. Dr. Merrick received the Peter Sabroe Child Award for outstanding work on behalf of Danish Children in 1985 and the International LEGO-Prize ("The Children's Nobel Prize") for an extraordinary contribution toward improvement in child welfare and well-being in 1987. Website: www.nichd–israel.com
